# Validation and optimization of AFP-based biomarker panels for early HCC detection in Latin America and Europe

**DOI:** 10.1097/HC9.0000000000000264

**Published:** 2023-09-15

**Authors:** Boris J.B. Beudeker, Siyu Fu, Domingo Balderramo, Angelo Z. Mattos, Enrique Carrera, Javier Diaz, Jhon Prieto, Jesus Banales, Arndt Vogel, Marco Arrese, Jeffrey Oliveira, Zwier M.A. Groothuismink, Gertine van Oord, Bettina E. Hansen, Robert A. de Man, José D. Debes, Andre Boonstra

**Affiliations:** 1 Department of Gastroenterology and Hepatology, Erasmus MC University Medical Center, Rotterdam, the Netherlands; 2 Department of Gastroenterology, Hospital Privado Universitario de Córdoba, Instituto Universitario de Ciencias Biomédicas de Córdoba, Córdoba, Argentina; 3 Graduate Program in Medicine: Hepatology, Federal University of Health Sciences of Porto Alegre, Porto Alegre, Brazil; 4 Departamento de Gastroenterologia y Hepatologia, Hospital Eugenio Espejo, Quito, Ecuador; 5 Department of Gastroenterology, Hospital Nacional Edgardo Rebagliati Martins, HNERM, Lima, Peru; 6 Centro de Enfermedades Hepáticas y Digestivas (CEHYD), Bogota, Colombia; 7 Department of Liver and Gastrointestinal Diseases, Biodonostia Health Research Institute, Donostia University Hospital, University of the Basque Country (UPV/EHU), CIBERehd, Ikerbasque, San Sebastian, Spain; 8 Department of Biochemistry and Genetics, School of Sciences, University of Navarra, Pamplona, Spain; 9 Department of Gastroenterology, Hepatology and Endocrinology, Hannover Medical School, Hannover, Germany; 10 Department of Gastroenterology, Pontificia Universidad Catolica de Chile, Santiago, Pontificia Universidad Catolica de Chile, Santiago, Chile; 11 Department of Epidemiology, Biostatistics, Erasmus MC University Medical Center, Rotterdam, the Netherlands, IHPME, University of Toronto & Toronto Center for Liver Disease, University Health Network, Toronto, Canada, Toronto; 12 Department of Medicine, University of Minnesota, Minneapolis, Minnesota, USA

## Abstract

**Background:**

HCC is a major cause of cancer death worldwide. Serum biomarkers such as alpha-fetoprotein (AFP), protein induced by vitamin K absence-II, and the Gender, Age, AFP-L3, AFP, Des-gamma-carboxy prothrombin (GALAD) score have been recommended for HCC surveillance. However, inconsistent recommendations in international guidelines limit their clinical utility.

**Methods:**

In this multicenter study, over 2000 patient samples were collected in 6 Latin American and 2 European countries. The performance of the GALAD score was validated in cirrhotic cases, and optimized versions were tested for early-stage HCC and prediagnostic HCC detection.

**Results:**

The GALAD score could distinguish between HCC and cirrhosis in Latin American patients with an AUC of 0.76, sensitivity of 70%, and specificity of 83% at the conventional cutoff value of −0.63. In a European cohort, GALAD had an AUC of 0.69, sensitivity of 66%, and specificity of 72%. Optimizing the score in the 2 large multicenter cohorts revealed that AFP-L3 contributed minimally to early-stage HCC detection. Thus, we developed a modified GALAD score without AFP-L3, the ASAP (age, sex, AFP, and protein induced by vitamin K absence-II), which showed promise for early-stage HCC detection upon validation. The ASAP score also identified patients with cirrhosis at high risk for advanced-stage HCC up to 15 months before diagnosis (*p* < 0.0001) and differentiated HCC from hemangiomas, with a specificity of 100% at 71% sensitivity.

**Conclusion:**

Our comprehensive analysis of large sample cohorts validates the GALAD score’s utility in Latin American, Spanish, and Dutch patients for early-stage HCC detection. The optimized GALAD without AFP-L3, the ASAP score, is a good alternative and shows greater promise for HCC prediction.

## Graphical abstract

**Figure:**
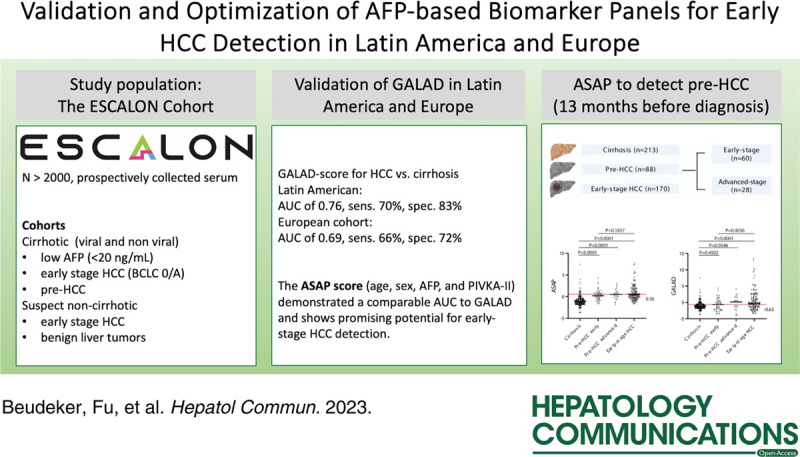

## INTRODUCTION

HCC is an aggressive form of liver cancer with poor prognosis.^1^ Early-stage detection of HCC is crucial for a successful outcome, as in later stages, a curative treatment might not be available. However, HCC often has no symptoms until it is advanced, leading to high mortality rates.^2^ This is particularly true in regions with reduced access to modern diagnostic imaging technology, such as Latin America.^3^ To address the high mortality rates of HCC, there is a need for reliable and accessible diagnostic tools that identify early-stage HCC in high-risk populations.^4^ HCC is primarily associated with cirrhosis, chronic HBV infection and NAFLD.^1^ Currently, individuals with risk factors, especially liver cirrhosis, are recommended to undergo surveillance with ultrasound imaging of the liver every 6 months, with optional alpha-fetoprotein (AFP) blood testing.^5^ However, the diagnostic accuracies of these methods are suboptimal, with ultrasonography’s sensitivity for detecting early-stage HCC being 47%, which increases to only 63% when combined with AFP.^6^ Efforts to identify more sensitive blood biomarkers for early-stage HCC detection are ongoing. The Gender, Age, AFP-L3, AFP, Des-gamma-carboxy prothrombin (DCP) (GALAD) score, which combines AFP lens culinaris agglutinin-reactive fraction of AFP (AFP-L3), and DCP [also known as protein induced by vitamin K absence or antagonist-II (PIVKA-II)] with age and gender, has demonstrated promising results in large, high-quality studies in the United Kingdom, United States, India, China, Japan, and Germany.^7–13^ Reported performance scores (area under the receiving operator curve [AUROC]) are high (0.93–0.98). In a large European cohort, at cutoff of −0.63, the GALAD score achieved a sensitivity of 92% and 90% specificity. However, control groups in some of these studies had lower rates of cirrhosis compared with HCC patients.^7,11–13^ Although chronic liver disease is a well-established risk factor for HCC, current biomarker screening methods are not suitable for individuals with no cirrhosis.^14^ Furthermore, cirrhosis-only cohorts associate with lower GALAD score AUROC values,^15,16^ highlighting the need for further confirmation studies in this population. Since HCC is a heterogeneous disease, validation of diagnostic tools such as the GALAD score in diverse patient populations is important to ensure their reliability and prioritize high specificity to prevent high false positive rates. It is currently unknown whether the GALAD score can effectively detect HCC in Latin American patients.

The objective of this study is to conduct a comprehensive evaluation of multiple biomarkers, including AFP, PIVKA-II, and the GALAD score, in well-characterized cohorts from both Europe and Latin America. By performing a detailed analysis of tumor stage, low serum AFP, liver disease etiologies, and pre-HCC diagnosis samples, we aim to provide an in-depth understanding of the applicability of these markers in patients with highly diverse HCC characteristics in these regions. Ultimately, our goal is to validate and support the implementation of these biomarkers in international guidelines, leading to better patient outcomes.

## METHODS

### Selection criteria of patient inclusion

The study evaluated tumor marker levels in serum from archived and prospectively collected samples from different cohorts in Europe and Latin America, described below and shown in Supplemental Figure S1, http://links.lww.com/HC9/A527. Patient identification and characterization included the diagnosis of HCC by histological and/or radiological evidence in accordance with European guidelines.^17^ Patients diagnosed with sufficient information on liver disease etiology and fibrosis status were included. Patients were excluded in cases of HCC recurrence, mixed-type HCC, non-HCC liver metastases, age < 18 years, co-existing non-HCC malignancies, and suspect non-cirrhotic HCC liver with elastography ranging from > 7.0 to < 12 kPa with missing pathology confirmation. Additional exclusion criteria were (1) current treatment with vitamin K inhibitors, which affect PIVKA-II serum levels; (2) pregnancy, which may influence serum AFP testing; (3) no serum sample available. The study was conducted in accordance with the Declaration of Helsinki and was approved by the local and/or national ethics committees in all participating centers. Written informed consent was obtained from all participants.

The serum samples were stored blinded to case and control status, and the levels of biomarkers were not made available or used for clinical decision-making. Detailed information on all patient samples and cohorts is presented in Supplemental Table S1, http://links.lww.com/HC9/A526 (Latin American centers) and Supplemental Table S2, http://links.lww.com/HC9/A526 (European centers).

### Patient cohorts

#### ESCALON cohort from Latin America and Europe

The patient cohort included individuals from both Latin America (Argentina, Peru, Brazil, Chile, Colombia, and Ecuador) and Europe (The Netherlands and Spain), who were enrolled in the ESCALON consortium (www.escalon.eu) since 2019. Cases and controls consisted of patients who were eligible for HCC surveillance and had cirrhosis. Details on etiology, tumor stage, and fibrosis assessment can be found in the Supplemental Tables, http://links.lww.com/HC9/A526. Sera were collected prospectively starting from 2019 and onwards and collected specifically for HCC biomarker discovery and validation. Patient information from collected samples in the ESCALON cohort was collected at the respective sites and registered in a Research Electronic Data Capture registry. All data was regularly checked by a data monitor and double-checked by the managing site before the analysis of the data. The control group was required to have a minimum follow-up of 12 months after biomarker assessment to confirm the absence of HCC. Sera samples from patients diagnosed with HCC were collected at the time of diagnosis.

#### Erasmus MC cirrhotic cohort (European cohort)

Cases and controls followed at the Erasmus Medical Center in the period 2009–2020 were retrospectively identified. Serum samples were collected during routine HCC surveillance visits and follow-ups of cirrhosis and HCC. Patient information was retrospectively obtained from the electronic medical records.

#### Suspect non-cirrhotic cohort (European cohort)

Patient information and sera were prospectively collected at Erasmus MC for all patients with no cirrhosis with elastography < 7 kpa who visited the outpatient clinic with a benign or malignant liver lesion over the course of 1 year.

#### HCC pre-diagnosis cohort (European cohort)

This cohort comprises patients with cirrhosis who underwent biannual HCC screening and had a serum sample collected between 8 and 16 months before HCC confirmation. They were compared with a control group of patients with cirrhosis who had a minimum follow-up of 24 months to ensure HCC exclusion.

### Assessment of etiology, tumor stage, and fibrosis

To determine the etiology, tumor stage, and fibrosis levels, medical records and confirmatory imaging, pathology, and laboratory tests were used. Patients with viral etiology were classified as having HBV, HCV, HCV/HBV, or HBV/HDV co-infection. Patients with alcohol-associated liver disease-related HCC had persistent steatohepatitis following an estimated daily ethanol intake of more than 40 g/day for men and more than 30 g/day for women for over 10 years, without other liver damage triggers. Patients with NAFLD-related HCC had either a diagnosis assigned by the managing hepatologists or evidence of hepatic steatosis by histopathology or ultrasound in the absence of alternative liver diseases. Other etiologies included acquired immune deficiencies, autoimmune liver disease, hemochromatosis, Wilson’s disease, alpha-1 antitrypsin deficiency, primary biliary cholangitis, primary sclerosing cholangitis, a documented clinical history of immunomodulatory drugs, pathology-proven Metavir F2-F3 fibrosis without risk factors, and monogenic syndromes predisposing to HCC.

Tumor stage evaluation: For patients with severe fibrosis and cirrhotic HCC, the Barcelona Clinic Liver Cancer (BCLC) staging system was used,^5^ categorizing the disease as early-stage (BCLC stage 0 and A), intermediate-stage (BCLC stage B), and advanced-stage (BCLC stage C and D). For suspect non-cirrhotic HCC, a modified version of the eighth TNM edition was used, categorizing the disease as early-stage (TNM stage T1–T2, eligible for curative treatment), intermediate-stage (TNM stage T3, eligible for treatment), and advanced-stage (T4 or not eligible for treatment due to poor World Health Organization performance status).

Fibrosis stage assessment: To determine the fibrosis stage of the patients, we considered either the stage at the time of HCC diagnosis or the stage that existed up to 1 year prior to the diagnosis. The presence of severe fibrosis or cirrhosis was established either by the managing hepatologists or through pathology (Metavir ≥ F3–F4) or liver transient elastography studies (> 12.0 kPa).

### Measurement of AFP and PIVKA-II using the Lumipulse analyzer

Serum samples were stored at −80°C, thawed, and subjected to measurement of AFP and PIVKA-II using the LUMIPULSE G1200 (Fujirebio, Tokyo, Japan). The assays were performed according to the instructions of the manufacturer. Serum AFP levels were determined in ng/ml using the LUMIPULSE G AFP-N kit and serum PIVKA-II levels in mAU/mL using the LUMIPULSE G PIVKA-II kit (Fujirebio Inc.). Low and high limits of detection for AFP were 0.5 and 2000 ng/ml, respectively, and for PIVKA-II, 5 and 75000 mAU/ml, respectively.

### Measurement of AFP, AFP-L3, and DCP using the Wako uTAS i30 analyzer

Serum samples were stored at −80°C, thawed and subjected to measurement of AFP, AFP-L3, and DCP using the μTAS Wako i30 fully automated immunoanalyzer (FUJIFILM Wako Chemicals Europe GmbH, Neuss, Germany). For assays, the μTAS Wako kits were performed according to the instructions of the manufacturer. Serum levels of AFP and DCP were in ng/ml, while AFP-L3 was expressed as the fraction of total AFP (as %). Low and high limits of detection were for AFP 0.3 and 2000 ng/ml, respectively, and for PIVKA-II 0.1 and 950 ng/ml, respectively. The GALAD score was calculated using the algorithm provided by the manufacturer and as described:^8^

Z=−10.08 + 1.67 × [Gender (1 for male, 0 for female)] + 0.09 × [Age] + 0.04 × [AFP-L3%] + 2.34 × log[AFP] + 1.33 × log[DCP]. If AFP-L3 (%) is below the detection limit, a default value of 0.5 (%) was given.

Both PIVKA-II and DCP are biomarkers that measure the same protein. To ensure consistency and clarity in this study, whenever DCP was mentioned, it was written as DCP(PIVKA-II) to indicate that it is measuring the same protein.

### Statistics

Statistical analyses were performed by SPSS 28.0.1.0 (SPSS Inc., an IBM Company, and Chicago, IL, USA). Categorical variables were presented as percentages and continuous variables as medians (interquartile ranges). Descriptive statistics were used to summarize the patient characteristics in the case and control groups. The Mann-Whitney *U* test was used for testing continuous variables, chi-square tests for dichotomous variables, and Spearman’s rank for correlation testing. The AUC of the ROC curves were used to determine the performance of the following biomarkers: GALAD, AFP, AFP-L3, PIVKA-II/DCP, and ASAP. The ROC curves were also used to identify the ideal cutoff values for each biomarker by applying the following strategy:

For 90% specificity, for the GALAD the cutoff of −0.63 was used.^8,10^ Similarly, for AFP the cutoff value of 20 ng/mL was used, which is recommended by the international guidelines.^17,18^ For AFP-L3; a cutoff value of 10%, PIVKA-II; the cutoff value of 40 mAU/mL, DCP (PIVKA-II); the cutoff value of 7.5 ng/mL were used.^8,10,15,17–20^ In addition, Youden’s index was used to identify the ideal cutoff values for the GALAD and ASAP scores that maximized both sensitivity and specificity.^21^ Sensitivity, specificity, and corresponding AUC at the cutoff were compared between the original and recalculated models. A 2-tailed value of *p* < 0.05 was considered statistically significant.

## RESULTS

### GALAD score performance in Latin American and European patients

**FIGURE 1:**
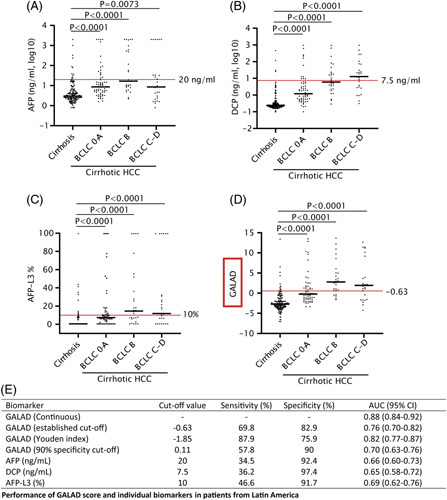
Performance of AFP, DCP (PIVKA-II), AFP-L3, and the GALAD score in HCC patients compared with patients with cirrhosis from Latin America. Most of the patients had biomarker levels below the cutoff value of AFP (A), DCP (PIVKA-II) (B), and AFP-L3 (C). The GALAD score (D) can distinguish more HCC patients compared with cirrhosis. Performance of the biomarkers (E). The Mann-Whitney *U* test was used to test the statistical difference. Abbreviations: AFP, alpha-fetoprotein; AFP-L3, lectin-reactive alpha-fetoprotein; DCP, Des-gamma-carboxy prothrombin; GALAD Score, Gender, Age, AFP-L3, AFP, DCP Score; PIVKA-II, protein induced by vitamin K absence-II.

To validate the efficacy of the GALAD score in Latin American individuals, we conducted a multicenter study using a cohort of 587 patients from 6 medical centers across 6 Latin American countries (Supplemental Table S1, http://links.lww.com/HC9/A526). These patients were specifically enrolled for HCC biomarker discovery and validation purposes. Blood samples were collected from all patients, who were then followed up for a minimum of 12 months. The study population comprised patients with cirrhosis who underwent biannual surveillance with liver imaging and AFP. By analyzing the data of 116 cirrhotic HCC versus 158 patients with cirrhosis, we found that the median values for AFP, DCP (PIVKA-II), and AFP-L3 were significantly higher in patients with HCC than in those with cirrhosis, especially in HCC patients with BCLC stage B, C and D (Figure 1). However, albeit significantly, the individual biomarkers AFP, DCP (PIVKA-II), and AFP-L3 poorly discriminated between HCC and cirrhosis, as measured by the AUCs for the established cutoffs between 0.65 and 0.69, and sensitivities between 35% and 47% (Figure 1E). The GALAD score demonstrated higher values in patients with HCC compared with those with cirrhosis, as illustrated in Figure 1. When applying the conventional cutoff value of −0.63, the GALAD score exhibited an AUC of 0.76, with a sensitivity of 70% and specificity of 83% (Figure 1E). As expected, the performance of the continuous GALAD score resulted in higher values (AUC=0.88) compared with the pre-defined cutoff (Figure 1E), which may suggest that a different cutoff may better differentiate HCC from non-HCC in our population: optimized cutoff −1.85; 88% sensitivity at 76% specificity. Interestingly, we found that the AUC of DCP (PIVKA-II) was performing a little better than that of GALAD (AUROC 0.90 vs. 0.88) for discriminating between HCC and cirrhosis. We also included samples of a European cirrhotic HCC cohort (Supplemental Table S2, http://links.lww.com/HC9/A526) and performed a similar analysis. In the European cohort, the original GALAD score at the conventional cutoff −0.63 had an AUC of 0.69 with 66% sensitivity and 72% specificity (Table 1, Supplemental Figure S2, http://links.lww.com/HC9/A528), while at the optimized cutoff of −1.62, it had sensitivity of 83% and specificity of 61%. At the optimized Latin American GALAD cutoff (−1.85), we found a higher sensitivity at the cost of specificity, 85% and 55%, respectively. Overall, as reported previously, the GALAD score is superior in distinguishing between HCC and cirrhosis when compared with traditional biomarkers, such as AFP, DCP (PIVKA-II), and AFP-L3. However, our results suggest that its performance may not be as high as reported for other cohorts; the specificity is generally low, and performance is impacted by geographical differences.

**TABLE 1:** Performance of GALAD score and individual biomarkers in patients from Europe

Biomarker	Cutoff value	Sensitivity (%)	Specificity (%)	AUC (95% CI)
GALAD (Continuous)	−	−	−	0.75 (0.70–0.81)
GALAD (established cutoff)	−0.63	65.8	71.7	0.69 (0.63–0.75)
GALAD (Youden index)	−1.62	82.6	60.6	0.71 (0.65–0.77)
GALAD (90% specificity cutoff)	1.72	32.9	90	0.62 (0.58–0.68)
AFP (ng/mL)	20	29.2	87.4	0.59 (0.52–0.65)
DCP (ng/mL)	7.5	26.7	87.4	0.57 (0.51–0.64)
AFP-L3 (%)	10	30.4	81.1	0.56 (0.50–0.63)

Abbreviations: AFP, alpha-fetoprotein; AFP-L3, lectin-reactive alpha-fetoprotein; DCP, Des-gamma-carboxy prothrombin; GALAD Score, Gender, Age, AFP-L3, AFP, DCP Score.

### Assessing the performance of the GALAD score in early-stage HCC patients from Latin America, and in patients with different etiologies

Next, we determined the performance of the GALAD score in subgroups of Latin American HCC patients with different AFP levels, tumor stages, and etiologies. First, we compared patients with cirrhosis (n=158) to patients with HCC with AFP levels below 20 ng/mL (n=76) and those with early-stage HCC (n=60). At the conventional cutoff of −0.63, patients with low serum AFP levels showed a GALAD score with an AUC of 0.68 (Supplemental Table S3A, http://links.lww.com/HC9/A526), and for early-stage HCC an AUC of 0.71 was found (Supplemental Table S3B, http://links.lww.com/HC9/A526). We also investigated the performance of the GALAD score in patients with cirrhosis caused by viral hepatitis and those with non-viral etiology. At the conventional cutoff of −0.63, GALAD had an AUC of 0.78 in patients with viral etiology and 0.75 in patients with non-viral etiology (Supplemental Tables S3C and S3D, http://links.lww.com/HC9/A526). In conclusion, our study provides the first evidence of the performance of the GALAD score for early-stage HCC detection in Latin American patients, and we show that further optimization of the GALAD score is required, especially for detecting small tumors or tumors with low AFP levels.

### Although the formula of AFP, AFP-L3, DCP (PIVKA-II), sex, and age-based score changes significantly when calculated using different cohorts, the performance is similar to the GALAD score when optimized cutoffs are used

The discovery of the GALAD score in 2014 represented a promising advancement in biomarker-based HCC detection.^8^ However, since then, no modifications or optimizations have been made to the GALAD formula, despite changes in the HCC patient population. To bridge this gap, we conducted an evaluation using our European and Latin American cohorts, where we refitted GALAD data points (gender, age, AFP, AFP-L3, and DCP (PIVKA-II)) into modified scores. Performance was tested in all our subjects with cirrhosis by determining the AUROC.

We developed 3 modified versions: European patients (model 1), Latin American patients (model 2), and only early-stage HCC (model 3). As presented in Supplemental Table S4, http://links.lww.com/HC9/A526, model 1, which adjusted the weight of age, gender, AFP-L3, and DCP (PIVKA-II), performed slightly better than the original GALAD score (AUROC 0.79 vs. 0.75). Model 2 also resulted in only a minor improvement in performance (AUROC 0.91 vs. 0.88). The modified GALAD model 3, with solely early-stage HCC and cirrhosis cases, performed similarly to the original GALAD score (AUROC 0.80 vs. 0.80), which was previously developed using all stages of HCC (Supplemental Table S4, http://links.lww.com/HC9/A526). Interestingly, this modified early-stage HCC-based score demonstrated no additional benefit of including AFP-L3 in the formula since the contribution of AFP-L3 to model 3 was 0.000. Even in models 1 and 2, the weight of AFP-L3 was very modest, which made us consider that leaving out AFP-L3 from a modified GALAD score would not or only minimally affect the performance.

### Modified GALAD, without AFP-L3, to detect early-stage HCC in the cirrhotic liver

As shown in Figure 1, early-stage HCC is associated with lower AFP levels. In addition, the detection of early-stage HCC using a modified GALAD score does not require AFP-L3. To enhance HCC surveillance, it is crucial to prioritize biomarker panels for patients with low AFP levels since AFP levels above 20 ng/mL usually trigger follow-up procedures. Consequently, we decided that excluding AFP-L3 from the modified GALAD formula was a reasonable step forward. Therefore, we conducted a new analysis to evaluate the impact of AFP-L3 exclusion. We analyzed European and Latin American patients with cirrhosis with HCC (n=744) and compared them with 584 cirrhotic controls (Supplemental Table S1, http://links.lww.com/HC9/A526 and Supplemental Table S2, http://links.lww.com/HC9/A526). Tumor stages were BCLC 0/A 511 (68.7%), BCLC B 126 (16.9%), BCLC C 59 (7.9%), and BCLC 42 (5.6%). As expected, AFP and PVIKA-II exhibited limited discriminatory ability when tested at established cutoffs for distinguishing between HCC and cirrhosis (AFP <20 ng/mL, AUC 0.72; PIVKA-II 40 mAU/ml, AUC 0.68) (Table 2).

**TABLE 2:** Performance of ASAP score and individual biomarkers in overall HCC and early-stage HCC patients compared with cirrhosis (EU+LA)

Biomarker	Cutoff value	Sensitivity (%)	Specificity (%)	AUC (95% CI)
**Overall HCC**				
ASAP (Continuous)	−	−	−	0.85 (0.83–0.87)
ASAP (Youden index)	0.56	70.6	84.9	0.76 (0.74–0.79)
ASAP (90% specificity cutoff)	0.66	61.4	90	0.76 (0.73–0.79)
AFP (ng/mL)	20	44.9	95.4	0.72 (0.69–0.74)
PIVKA-II (mAU/mL)	40	88.3	39.2	0.68 (0.66–0.71)
Early-stage HCC				
ASAP (Continuous)	−	−	−	0.82 (0.79–0.84)
ASAP (Youden index)	0.21	64.6	83.7	0.74 (0.71–0.77)
ASAP (90% specificity cutoff)	0.70	52.8	90	0.71 (0.68–0.75)
AFP (ng/mL)	20	38.6	95.4	0.67 (0.64–0.70)
PIVKA-II (mAU/mL)	40	85.3	39.2	0.62 (0.59–0.65)

Abbreviations: AFP, alpha-fetoprotein; AFP-L3, lectin-reactive alpha-fetoprotein; ASAP, age, sex, AFP, and PIVKA-II; DCP, Des-gamma-carboxy prothrombin; GALAD Score, Gender, Age, AFP-L3, AFP, DCP Score; PIVKA-II, protein induced by vitamin K absence-II.

Next, we developed a new score using European and Latin American patients, which included log(PIVKA-II), log(AFP), and age and sex (ASAP) (Supplemental Table S5A, http://links.lww.com/HC9/A526). Other factors were not included in the logistic regression analysis since we aimed to test a model solely based on gender, age, AFP, and PIVKA-II levels. The formula for the ASAP model is as follows: Z= −6.836 + 0.042 × age + 0.989 × gender + 1.841 × log10(AFP) + 0.949 × log10(PIVKA-II) (Supplemental Table S5B, http://links.lww.com/HC9/A526). The ASAP model had an AUROC of 0.85 for discriminating cirrhotic HCC from patients with cirrhosis. At 90% specificity, the sensitivity was 61% (Table 2), and for early-stage HCC AUROC, 0.82 (53% sensitivity, 90% specificity) (Table 2).

### ASAP shows excellent performance following validation in cirrhotic and suspect non-cirrhotic cases

Next, we validated the performance of ASAP in European and Latin American ASAP cohorts separately: The ASAP AUROC for European all-stage versus patients with cirrhosis was 0.83 (59% sensitivity, 90% specificity), and for Latin American patients, 0.89 (72% sensitivity, 90% specificity). When considering only early-stage HCC, then the AUROC for ASAP of European samples was 0.80 (52% sensitivity, 90% specificity), and for Latin American patients, 0.86 (63% sensitivity, 90% specificity).

**FIGURE 2:**
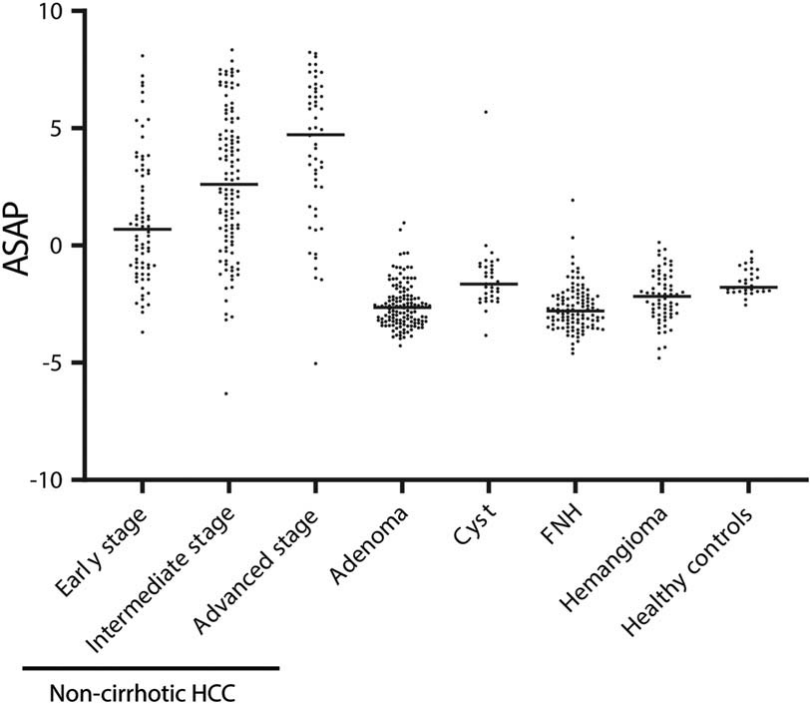
Performance of the ASAP score in the suspect non-cirrhotic HCC group compared with healthy individuals with benign tumors. The ASAP score showed an increasing trend from early-stage HCC to late-stage HCC, presenting good performance in distinguishing non-cirrhotic HCC from non-cancer groups. Abbreviations: ASAP, age, sex, AFP, and PIVKA-II.

To investigate whether ASAP could also be used to differentiate HCC from benign liver tumors in non-cirrhotic background, we analyzed serum samples from individuals with non-cirrhotic HCC (n=241) and benign tumors such as adenomatosis (n=136) or hemangiomas (n=64) (Supplemental Table S6, http://links.lww.com/HC9/A526). As shown in Figure 2, the ASAP model showed an excellent AUROC of 0.94 for discriminating HCC from benign liver tumors, including adenomas, focal nodular hyperplasia (FNH), cysts, and hemangiomas. Furthermore, ASAP exhibited an AUROC of 0.88 and 85.5% sensitivity at 90% specificity in discriminating early-stage HCC from benign tumors. Notably, at a cutoff value of 0.18, ASAP demonstrated 100% specificity (73% sensitivity) in distinguishing HCC from vascular tumor hemangioma, which typically requires contrast-enhanced imaging for proper differentiation.

### GALAD and ASAP show comparable performance in discriminating cirrhosis from early-stage HCC

To compare the performance of ASAP and GALAD in discriminating cirrhosis from early-stage HCC, we first evaluated the correlation and discriminatory performance between the 2 models. The results of the correlation analysis showed a near-perfect correlation between the 2 scores (r=0.96, *p* < 0.0001) (Supplemental Figure S3A, http://links.lww.com/HC9/A529). As expected, the PIVKA-II levels determined on the Lumipulse instrument and DCP levels determined on the uTAS i30 analyzer were highly correlated (*r*=0.97, *p* < 0.0001; Supplemental Figure S3B, http://links.lww.com/HC9/A530).

We then compared the performance of the ASAP and GALAD scores in a combined cohort of patients who had both measurements available (Supplemental Table S1, http://links.lww.com/HC9/A526 and Supplemental S2, http://links.lww.com/HC9/A526). The analysis showed that the ASAP and GALAD scores had comparable AUROCs for the detection of early-stage HCC, with minimal difference between the 2 scores (AUROC ASAP early-stage HCC: AUROC 0.85, 54.1% sensitivity, 90% specificity, and AUROC GALAD early-stage HCC: AUROC 0.85, 48.8% sensitivity, 90% specificity, Table 3). This finding suggests that the ASAP score, which was designed for HCC detection in patients with low AFP levels, is as effective as GALAD in discriminating early-stage HCC from cirrhosis, a finding that requires further validation in prospectively designed trials.

**TABLE 3:** Comparison of GALAD, ASAP and individual biomarkers in patients with early-stage HCC

Biomarker	Cutoff value	Sensitivity (%)	Specificity (%)	AUC (95% CI)
GALAD (Continuous)	−	−	−	0.85 (0.82–0.89)
GALAD	-0.63	61	86	0.74 (0.68–0.79)
ASAP (Continuous)	−	−	−	0.85 (0.81–0.89)
ASAP	0.56	45	91	0.68 (0.63–0.74)
AFP (ng/mL)	20	23	95	0.59 (0.53–0.65)
DCP (ng/mL)	7.5	18	95	0.57 (0.51–0.62)
AFP-L3 (%)	10	25	92	0.58 (0.52–0.64)
PIVKA-II (mAU/mL)	40	88	62	0.75 (0.70–0.79)

Abbreviations: Abbreviations: AFP, alpha-fetoprotein; AFP-L3, lectin-reactive alpha-fetoprotein; ASAP, age, sex, AFP, and PIVKA-II; DCP, Des-gamma-carboxy prothrombin; GALAD Score, Gender, Age, AFP-L3, AFP, DCP Score; PIVKA-II, protein induced by vitamin K absence-II.

### The ASAP and GALAD scores identify patients at high risk for advanced HCC development in an average of 13 months.

**FIGURE 3:**
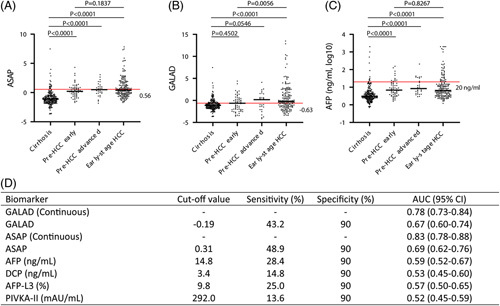
Performance of ASAP (A), GALAD (B), and AFP (C) in pre-HCC samples compared with patients with cirrhosis who did not develop HCC at follow-up. Performance of the biomarkers (D). The ASAP score distinguishes pre-HCC [13 (SD 3.4) mo before diagnosis] samples from patients who eventually are diagnosed with early-stage HCC from cirrhosis (A, *p* < 0.05), while GALAD only showed a difference between cirrhotic samples and pre-HCC samples from patients who eventually are diagnosed with advanced HCC (B, *p* > 0.05). Although AFP exhibited higher levels in pre-HCC samples in the early-stage diagnosed group compared with cirrhosis (C, *p* < 0.05), most of the patients had lower AFP levels than the cutoff value. Abbreviations: AFP, alpha-fetoprotein; ASAP, age, sex, AFP, and PIVKA-II; GALAD Score, Gender, Age, AFP-L3, AFP, DCP Score.

Given the increasing interest in predictive biomarkers suitable for screening efforts, we next conducted an analysis of 313 patients with cirrhosis enrolled in HCC surveillance, with at least 24 months of follow-up and compared them to 88 cases that developed HCC (Supplemental Table S7, http://links.lww.com/HC9/A526). We determined the ASAP and GALAD scores in sera collected in an average of 13 (SD 3.4) months before HCC diagnosis, as confirmed by contrast-enhanced imaging. In addition, we included 170 patients with cirrhosis with early-stage HCC as a reference group. Our analysis found that AFP (AUROC 0.59 and 28.4% sensitivity, 90% specificity), AFP-L3 (AUROC 0.57 and 25.0% sensitivity, 90% specificity), PIVKA-II (AUROC 0.52 and 13.6% sensitivity, 90% specificity), and DCP (PIVKA-II) (AUROC 0.53 and 14.8% sensitivity, 90% specificity) did not discriminate pre-HCC from cirrhosis well. In contrast, ASAP had an AUROC of 0.83, and GALAD had an AUROC of 0.78 (Figure 3D). Interestingly, ASAP was able to discriminate cirrhosis from both early-stage and advanced-stage pre-HCC (Figure 3A, *p* < 0.0001 for both comparisons), while GALAD only showed a trend for discriminating cirrhosis from advanced-stage pre-HCC (*p* > 0.05) (Figure 3B). These findings suggest that ASAP may be a useful tool in identifying patients with a high risk for aggressive HCC who may benefit from more rigorous screening or liver transplantation. However, additional studies are warranted to thoroughly assess the clinical utility of ASAP and GALAD scores in terms of screening efficacy and their ability to predict tumor aggressiveness.

## DISCUSSION

In the current study, we validated the performance of the GALAD model in detecting HCC in Latin American, Spanish, and Dutch patients with cirrhosis. The model has initially been developed to predict the likelihood of individual patients with cirrhosis having HCC, and we confirm that the score is superior to AFP alone. Our study demonstrates that the GALAD's performance in detecting all-stage HCC is lower than that of the 2014 study, with an AUROC of 0.88 in Latin American patients compared with 0.97, respectively. This difference in performance could be attributed to the possibility of overfitting the specific data used, highlighting the importance of evaluating models on new datasets. In addition, in our Latin American and European cohorts, the conventional cutoff of −0.63 did not associate with 90% specificity nor optimal sensitivity. The difference in performance is likely due to the low sensitivity and specificity of AFP in our patients. Other GALAD studies give figures of > 0.87 for AUROC of AFP-based all-stage HCC detection, while we observe AUC < 0.65.^7–13^ These differences might be explained by the high frequency of early-stage HCC cases in our cohort, low rates of HCV-associated HCC, balanced control groups with similar frequencies of cirrhosis, and population genetics. Moreover, our analysis comparing diagnostic AFP levels with stored serum sample AFP values revealed a near-perfect correlation (*R*=0.92, *p* < 0.0001), confirming the accuracy and reliability of our AFP measurement.

In our ASAP score, we also evaluated the performance of DCP instead of PIVKA-II in a small number of samples, showing similar performance for both markers (early-stage HCC (AUROC ASAP (PIVKA-II) vs. ASAP (DCP): 0.841 vs. 0.845 respectively)). The near-perfect correlation between PIVKA-II and DCP, along with their comparable performance, suggests their interchangeable use within the ASAP score.

We evaluated the modified GALAD models 1 to 3 by incorporating GALAD data points. Remarkably, the data points differed for each model. However, irrespective of the assigned weights to the formula components, the models exhibited similar performance. This suggests that the predictive capability of the GALAD score primarily depends on the presence and combination of specific biomarkers rather than the specific weights assigned to them.

Upon conducting the logistic regression analyses, we discovered that the inclusion of AFP-L3 did not significantly improve the modified GALAD score’s capacity for optimizing early-stage HCC detection. Subsequently, we developed a novel AFP-based model, named ASAP (age, sex, (log)AFP, (log)PIVKA-II), which performed equally well as the GALAD score in detecting low AFP early-stage HCC and showed even greater potential for very early HCC detection, up to 13 months before diagnosis. Our study, with a larger sample size and an average pre-diagnosis sample period of 13 (SD 3.4) months, offers a significant advantage over previous studies, providing more robust evidence of the effectiveness of the GALAD and ASAP scores in predicting advanced HCC development in patients with cirrhosis.^16^ In an earlier analysis of samples primarily from the early 2000s with HCV-associated HCC cases, a sensitivity of up to 66% at 90% specificity was reported. However, it is important to note that the effectiveness of the GALAD score in this analysis was likely predominantly driven by AFP.^15^

Our study is the first to differentiate between patients who will develop early-stage or advanced HCC. We found that higher GALAD or ASAP scores in pre-diagnosis samples were strongly associated with more aggressive disease. In this light, our study demonstrated that the GALAD and ASAP scores can complement conventional methods in identifying small or hypovascular HCC that may have been missed with high-resolution imaging or identifying patients that require more rigorous follow-up of their cirrhosis.

We observed excellent performance for the ASAP score in the context of suspect non-cirrhotic HCC, elastography < 7 kPa. The findings suggest that the ASAP score has the potential to complement clinical decision-making and differentiate malignant from benign diseases. We acknowledge that screening for HCC in individuals with no cirrhosis may not be feasible due to the low rate of HCC occurrence in this group. Based on our data, we strongly suggest that the GALAD score be incorporated into HCC early-detection protocols with a cutoff at 90% specificity, which should be tailored to the population being screened. Lastly, for cost-effective HCC screening in hospitals, consider incorporating an AFP, sex, age, and PIVKA-II-based approach. This combination is financially viable, utilizing the widely used AFP test with just 1 additional measurement. It is particularly suitable for low-resource settings or hospitals with limited AFP-L3 measurement capabilities.

## Supplementary Material
