# Supplementary material for: Validation and optimization of AFP-based biomarker panels for early HCC detection in Latin America and Europe
Source: Hepatol Commun. 2023 Sep 15;7(10):e0264. doi: 10.1097/HC9.0000000000000264 (PMC10503685; doi:10.1097/HC9.0000000000000264)
Supplement: Supplementary file 1 [file hc9-7-e0264-s001.docx]

**Table S1. Characteristics of Latin American patients.**

|  | ASAP | | GALAD | | ASAP/GALAD overlapping | |
| --- | --- | --- | --- | --- | --- | --- |
| Variable | HCC (cirrhotic) | Control (cirrhotic) | HCC (cirrhotic) | Control (cirrhotic) | HCC (cirrhotic) | Control (cirrhotic) |
| Number | 188 | 393 | 116 | 158 | 113 | 155 |
| Male | 124 (66.0%) | 204 (51.9%) | 76 (65.5%) | 77 (48.7%) | 75 (66.4%) | 76 (49.0%) |
| Age | 68 (62 to 73) | 63 (58 to 69) | 67 (61 to 72) | 65 (59 to 70) | 66 (60 to 72) | 65 (59 to 70) |
| AFP (ng/ml) | 26.6 (5.8 to 1012.6) | 4.3 (2.9 to 6.4) | 10.6 (4.6 to 50.4) | 3.0 (2.2 to 5.3) | 10.6 (4.6 to 54.3) | 3.0 (2.2 to 5.3) |
| PIVKA-II (mAU/ml) | 574.5 (110.5 to 7154.0) | 47.0 (33.0 to 110.5) | NA | NA | 339.0 (84.5 to 1827.5) | 27.0 (23.0 to 36.0) |
| DCP (ng/ml) | NA | NA | 3.2 (0.9 to 17.5) | 0.2 (0.2 to 0.3) | 3.1 (0.9 to 17.0) | 0.2 (0.2 to 0.3) |
| AFP-L3 (%) | NA | NA | 9.5 (3.2 to 26.3) | 0.5 (0.5 to 5.1) | 9.7 (3.5 to 27.2) | 0.5 (0.5 to 4.9) |
| ASAP | 1.99 (0.47 to 5.22) | -0.77 (-1.31 to -0.16) | NA | NA | 1.21 (-0.14 to 2.66) | -1.19 (-1.67 to -0.57) |
| GALAD | NA | NA | 1.00 (-1.12 to 3.31) | -2.71 (-3.48 to -1.86) | 1.02 (-1.10 to 3.52) | -2.71 (-3.46 to -1.96) |
|  |  |  |  |  |  |  |
| **Stages, %** |  |  |  |  |  |  |
| Early (BCLC 0-A) | 91 (48.4%) | NA | 60 (51.7%) | NA | 59 (52.2%) | NA |
| Intermediate (BCLC B) | 43 (22.9%) | NA | 28 (24.1%) | NA | 26 (23.0%) | NA |
| Advanced (BCLC C-D) | 54 (28.7%) | NA | 27 (23.3%) | NA | 27 (23.9%) | NA |
| Unknown | 0 | NA | 1 (0.9%) | NA | 1 (0.9%) | NA |
|  |  |  |  |  |  |  |
| **Etiology, %** |  |  |  |  |  |  |
| HBV | 4 (2.1%) | 8 (2.0%) | 5 (4.3%) | 0 | 4 (3.5%) | 0 |
| HCV | 23 (12.2%) | 32 (8.1%) | 19 (16.4%) | 22 (13.9%) | 14 (12.4%) | 14 (9.0%) |
| NAFLD | 89 (47.3%) | 197 (50.1%) | 55 (47.4%) | 91 (57.6%) | 52 (46.0%) | 87 (56.1%) |
| ALD | 23 (12.2%) | 62 (15.8%) | 26 (22.4%) | 17 (10.8%) | 15 (13.3%) | 16 (10.3%) |
| Others | 49 (26.1%) | 94 (23.9%) | 11 (9.5%) | 28 (17.7%) | 28 (24.8%) | 38 (24.5%) |

Abbreviations: alpha-fetoprotein (AFP); lectin-reactive alpha-fetoprotein (AFP-L3); protein induced by vitamin K absence-II (PIVKA-II); Des-gamma-carboxy prothrombin (DCP); age, sex, AFP, and PIVKA-II (ASAP); Gender, Age, AFP-L3, AFP, DCP (GALAD); Barcelona clinic liver cancer staging (BCLC); hepatitis B virus (HBV); hepatitis C virus (HCV); alcoholic liver disease (ALD); non-alcoholic fatty liver disease (NAFLD).

**Table S2. Patient Characteristics in Europe.**

|  | ASAP | | GALAD | | ASAP/GALAD overlapping | |
| --- | --- | --- | --- | --- | --- | --- |
| Variable | HCC (cirrhotic) | Control (cirrhotic) | HCC (cirrhotic) | Control (cirrhotic) | HCC (cirrhotic) | Control (cirrhotic) |
| Number, n | 556 | 191 | 161 | 127 | 116 | 66 |
| Male, % | 440 (79.1%) | 145 (75.9%) | 127 (78.9%) | 102 (80.3%) | 91 (78.4%) | 50 (75.8%) |
| Age | 63 (56 to 70) | 54 (46 to 62) | 64 (56 to 73) | 57 (49 to 63) | 66 (59 to 74) | 56 (48 to 62) |
| AFP (ng/ml) | 13.1 (5.2 to 104.3) | 5.2 (3.3 to 8.9) | 7.9 (3.7 to 28.6) | 4.5 (3.2 to 8.7) | 6.5 (3.4 to 16.8) | 3.7 (2.6 to 5.2) |
| PIVKA-II (mAU/ml) | 179.0 (61.0 to 1205.5) | 51.0 (35.0 to 97.0) | NA | NA | 177.5 (62.3 to 463.3) | 60.0 (42.0 to 172.0) |
| DCP (ng/ml) | NA | NA | 1.9 (0.6 to 10.4) | 0.7 (0.3 to 2.2) | 1.6 (0.6 to 5.3) | 0.5 (0.3 to 2.0) |
| AFP-L3 (%) | NA | NA | 6.5 (0.5 to 15.8) | 0.5 (0.5 to 8.3) | 5.7 (0.5 to 5.3) | 0.5 (0.5 to 7.0) |
| ASAP | 1.05 (-0.06 to 2.89) | -0.72 (-1.29 to 0.03) | NA | NA | 0.49 (-0.22 to 1.66) | -0.82 (-1.56 to -0.41) |
| GALAD | NA | NA | 0.03 (-1.23 to 2.65) | -2.04 (-3.12 to -0.18) | -0.23 (-1.27 to 2.06) | -2.44 (-3.51 to -1.57) |
|  |  |  |  |  |  |  |
| **Stages, %** |  |  |  |  |  |  |
| Early (BCLC 0-A) | 420 (75.5%) | NA | 134 (83.2%) | NA | 111 (95.7%) | NA |
| Intermediate (BCLC B) | 83 (14.9%) | NA | 11 (6.8%) | NA | 3 (2.6%) | NA |
| Advanced (BCLC C-D) | 47 (8.4%) | NA | 11 (6.8%) | NA | 2 (1.7%) | NA |
| Unknown | 6 (1.1%) | NA | 5 (3.1%) | NA | 0 | NA |
|  |  |  |  |  |  |  |
| **Etiology, %** |  |  |  |  |  |  |
| HBV | 92 (16.5%) | 44 (22.9%) | 26 (16.1%) | 37 (29.1%) | 0 | 0 |
| HCV | 132 (23.7%) | 46 (24.0%) | 16 (9.9%) | 1 (0.8%) | 12 (10.3%) | 0 |
| NAFLD | 83 (14.9%) | 46 (24.0%) | 46 (28.6%) | 46 (36.2%) | 45 (38.8%) | 36 (54.5%) |
| ALD | 152 (27.3%) | 44 (22.9%) | 46 (28.6%) | 37 (29.1%) | 42 (36.2%) | 30 (45.5%) |
| Others | 97 (17.4%) | 12 (6.3%) | 27 (16.8%) | 6 (4.7%) | 17 (14.7%) | 0 |

Abbreviations: alpha-fetoprotein (AFP); lectin-reactive alpha-fetoprotein (AFP-L3); protein induced by vitamin K absence-II (PIVKA-II); Des-gamma-carboxy prothrombin (DCP); age, sex, AFP, and PIVKA-II (ASAP); Gender, Age, AFP-L3, AFP, DCP (GALAD); Barcelona clinic liver cancer staging (BCLC); hepatitis B virus (HBV); hepatitis C virus (HCV); alcoholic liver disease (ALD); non-alcoholic fatty liver disease (NAFLD)

**Table S3A. GALAD in low-AFP HCC Latin American cohort.**

| Biomarker | Cut-off value | Sensitivity (%) | Specificity (%) | AUC (95% CI) |
| --- | --- | --- | --- | --- |
| GALAD (continuous) | - | - | - | 0.83 (0.77-0.88) |
| GALAD (established cut-off) | -0.63 | 53.9 | 82.9 | 0.68 (0.61-0.76) |
| GALAD (Youden index) | -2.03 | 85.5 | 72.8 | 0.79 (0.73-0.85) |
| GALAD (90% specificity cut-off) | 0.11 | 38.2 | 90 | 0.64 (0.56-0.72) |
| AFP (ng/mL) | 20 | 0 | 92.4 | 0.46 (0.39-0.54) |
| DCP (ng/mL) | 7.5 | 30.3 | 97.5 | 0.64 (0.56-0.72) |
| AFP-L3 (%) | 10 | 34.2 | 91.8 | 0.63 (0.55-0.71) |

In our Latin American cohort, a total of 76 patients with cirrhotic hepatocellular carcinoma (HCC) and AFP levels below 20 ng/mL were compared to 158 patients with cirrhotic non-HCC livers. The ROC curve (receiver operating characteristic curve) was used to evaluate the performance of a classification model for continuous variables. The cut-off points for GALAD were determined based on a previous study (established cut-off), the optimum cut-off point (Youden index), and a 90% specificity cut-off. The cut-off values for AFP, DCP, and AFP-L3 were derived from relevant guidelines or literature sources (methods). Abbreviations: Alpha-fetoprotein (AFP); lectin-reactive alpha-fetoprotein (AFP-L3); Des-gamma-carboxy prothrombin (DCP); Gender, Age, AFP-L3, AFP, DCP (GALAD).

**Table S3B. GALAD score in early-stage HCC determined in the Latin American cohort.**

| Biomarker | Cut-off value | Sensitivity (%) | Specificity (%) | | AUC (95% CI) |
| --- | --- | --- | --- | --- | --- |
| GALAD (Continuous) | - | - | - | 0.84 (0.79-0.90) | |
| GALAD (established cut-off) | -0.63 | 58.3 | 82.9 | 0.71 (0.62-0.79) | |
| GALAD (Youden index) | -2.45 | 96.7 | 63.3 | 0.80 (0.74-0.86) | |
| GALAD (90% specificity cut-off) | 0.11 | 40.0 | 90 | 0.65 (0.57-0.74) | |
| AFP (ng/mL) | 20 | 26.7 | 92.4 | 0.60 (0.51-0.69) | |
| DCP (ng/mL) | 7.5 | 18.3 | 97.5 | 0.60 (0.49-0.67) | |
| AFP-L3 (%) | 10 | 35.0 | 91.8 | 0.63 (0.54-0.72) | |

In our Latin American cohort, a total of 60 patients with early-stage cirrhotic hepatocellular carcinoma (HCC) were compared to 158 patients with cirrhotic non-HCC livers. The ROC curve (receiver operating characteristic curve) was used to evaluate the performance of a classification model for continuous variables. The cut-off points for GALAD were determined based on a previous study (established cut-off), the optimum cut-off point (Youden index), and a 90% specificity cut-off. The cut-off values for AFP, DCP, and AFP-L3 were derived from relevant guidelines or literature sources (methods). Abbreviations: Alpha-fetoprotein (AFP); lectin-reactive alpha-fetoprotein (AFP-L3); Des-gamma-carboxy prothrombin (DCP); Gender, Age, AFP-L3, AFP, DCP (GALAD).

**Table S3C. GALAD score in viral HCC versus viral cirrhosis as determined in samples from the Latin American cohort.**

| Biomarker | Cut-off value | Sensitivity (%) | Specificity (%) | AUC (95% CI) |
| --- | --- | --- | --- | --- |
| GALAD (Continuous) | - | - | - | 0.89 (0.80-0.99) |
| GALAD (established cut-off) | -0.63 | 87.5 | 77.3 | 0.78 (0.64-0.92) |
| GALAD (Youden index) | -0.34 | 87.5 | 81.8 | 0.85 (0.73-0.97) |
| GALAD (90% specificity cut-off) | 0.62 | 70.8 | 90 | 0.81 (0.68-0.94) |
| AFP (ng/mL) | 20 | 37.5 | 81.8 | 0.62 (0.45-0.78) |
| DCP (ng/mL) | 7.5 | 33.3 | 95.5 | 0.64 (0.48-0.80) |
| AFP-L3 (%) | 10 | 62.5 | 90.9 | 0.77 (0.63-0.91) |

In our Latin American cohort, 24 cirrhotic HCC patients with HBV or HCV infection were compared to 22 samples from patients with cirrhotic viral non-HCC livers. The ROC curve (receiver operating characteristic curve) was used to evaluate the performance of a classification model for continuous variables. The cut-off points for GALAD were determined based on a previous study (established cut-off), the optimum cut-off point (Youden index), and a 90% specificity cut-off. The cut-off values for AFP, DCP, and AFP-L3 were derived from relevant guidelines or literature sources (methods). Abbreviations: Alpha-fetoprotein (AFP); lectin-reactive alpha-fetoprotein (AFP-L3); Des-gamma-carboxy prothrombin (DCP); Gender, Age, AFP-L3, AFP, DCP (GALAD).

**Table S3D. GALAD score in non-viral HCC versus non-viral cirrhosis as determined in samples from the Latin America cohort.**

| Biomarker | Cut-off value | Sensitivity, % | Specificity, % | AUC (95% CI) |
| --- | --- | --- | --- | --- |
| GALAD (Continuous) | - | - | - | 0.86 (0.80-0.91) |
| GALAD (established cut-off) | -0.63 | 67.9 | 82.4 | 0.75 (0.68-0.83) |
| GALAD (Youden index) | -1.85 | 85.2 | 75.0 | 0.80 (0.74-0.87) |
| GALAD (90% specificity cut-off) | 0.25 | 55.6 | 90 | 0.73 (0.66-0.81) |
| AFP (ng/mL) | 20 | 34.6 | 92.6 | 0.64 (0.55-0.72) |
| DCP (ng/mL) | 7.5 | 38.3 | 97.2 | 0.67 (0.59-0.75) |
| AFP-L3 (%) | 10 | 46.9 | 90.7 | 0.68 (0.60-0.76) |

In this cohort, 81 cirrhotic non-viral HCC (NAFLD or ALD) were compared to 108 corresponding patients with non-HCC cirrhotic livers from Latin America. The ROC curve (receiver operating characteristic curve) was used to evaluate the performance of a classification model for continuous variables. The cut-off points for GALAD were determined based on a previous study (established cut-off), the optimum cut-off point (Youden index), and a 90% specificity cut-off. The cut-off values for AFP, DCP, and AFP-L3 were derived from relevant guidelines or literature sources (methods). Abbreviations: Alpha-fetoprotein (AFP); lectin-reactive alpha-fetoprotein (AFP-L3); Des-gamma-carboxy prothrombin (DCP); Gender, Age, AFP-L3, AFP, DCP (GALAD).

**Table S4. Recalculated GALAD model in HCC patients from Latin America and Europe.**

| **Model** | **LA cohort, AUC** | **EU cohort, AUC** | **Early-stage HCC cohort (EU+LA), AUC** | **Formula** |
| --- | --- | --- | --- | --- |
| GALAD | 0.88 | 0.75 | 0.80 | -10.08 + 0.09 * age + 1.67 * gender + 2.34 * log(AFP) + 0.04 * AFP-L3 + 1.33 * log(DCP) |
| Model 1 | 0.79 | 0.79 | 0.76 | -5.75 + 0.082 * age + 0.086 * gender + 0.930 * log(AFP) + 0.004 * AFP-L3 + 0.381 * log(DCP) |
| Model 2 | 0.91 | 0.71 | 0.79 | -2.433 + 0.021 * age + 0.474 * gender + 0.600 * log(AFP) + 0.009 * AFP-L3 + 1.952 * log(DCP) |
| Model 3 | 0.86 | 0.77 | 0.80 | -5.468 + 0.059 * age + 0.681 * gender + 1.028 * log(AFP) + 0.000 * AFP-L3 + 0.811 * log(DCP) |

Cohort information was described in Table S1 and Table S2. Logistic regression was performed to calculate Model 1, 2, and 3 using the respective cohorts as presented in Suppl Table 1 and 2. Abbreviations: Gender, Age, AFP-L3, AFP, DCP (GALAD); Latin America (LA); Europe (EU).

**Table S5A. Cohort information for ASAP model development.**

| ASAP | Calculated by | Cohort | Number |
| --- | --- | --- | --- |
| Model 1 | Cohort 1 | Latin American cohort | 188 cirrhotic-HCC, 393 cirrhosis |
| Model 2 | Cohort 2 | European cohort | 556 cirrhotic-HCC, 191 cirrhosis |
| Model 3 | Cohort 3 | Latin American cohort  European cohort | 744 cirrhotic-HCC, 584 cirrhosis |
| Model 4 | Cohort 4 | Latin American cohort  European cohort | 744 cirrhotic-HCC, 584 cirrhosis, 241 non-cirrhotic HCC, 136 adenoma, 34 cysts, 113 FNH, 64 hemangioma, 31 healthy controls |

In our European and Latin American cohort, 188 cirrhotic-HCC and 393 cirrhosis from Latin America were used to develop cohort 1; 556 cirrhotic-HCC and 191 cirrhosis from Europe were used to develop cohort 2; 744 cirrhotic-HCC and 584 cirrhosis from Europe and Latin America were used to develop cohort 3; All cirrhotic HCC, non-cirrhotic HCC, cirrhotic controls, and non-cirrhotic controls from Europe and Latin America were used to develop cohort 4. Logistic regression was performed to calculate Model 1, 2, 3, and 4 with the respective cohorts as presented in the table. Abbreviations: alpha-fetoprotein (AFP); protein induced by vitamin K absence-II (PIVKA-II); age, sex, AFP, and PIVKA-II (ASAP).

**Table S5B. ASAP formula and its performance in different cohorts.**

| **Model** | **Cohort 1, AUC** | **Cohort 2, AUC** | **Cohort3, AUC** | **Cohort4, AUC** | **Formula** |
| --- | --- | --- | --- | --- | --- |
| Model 1 | 0.89 | 0.84 | 0.84 | 0.89 | -8.950 + 0.051 * age + 0.485 * gender + 1.942 * log(AFP) + 1.209 * log(PIVKA-II) |
| Model 2 | 0.88 | 0.85 | 0.83 | 0.88 | -7.787 + 0.086 * age + 0.499 * gender + 1.391 * log(AFP) + 0.945 * log(PIVKA-II) |
| Model 3 | 0.89 | 0.83 | 0.85 | 0.89 | -6.836 + 0.042 * age + 0.989 * gender + 1.841 * log(AFP) + 0.949 * log(PIVKA-II) |
| Model 4 | 0.89 | 0.83 | 0.85 | 0.89 | -7.228 + 0.045 * age + 1.118 * gender + 1.528 * log(AFP) + 1.161 * log(PIVKA-II) |

Cohort information was described in Table S5A. Logistic regression was performed to calculate Model 1, 2, 3, and 4 using the respective cohorts as presented in Suppl Table 5A. Abbreviations: alpha-fetoprotein (AFP); protein induced by vitamin K absence-II (PIVKA-II); age, sex, AFP, and PIVKA-II (ASAP).

**Table S6. Cohort information for non-cirrhotic HCC and benign liver occupying space disease.**

| Variables | HCC (non-cirrhotic) | Adenoma | Cyst | FNH | Hemangioma | Healthy controls |
| --- | --- | --- | --- | --- | --- | --- |
| Number, n | 241 | 136 | 34 | 113 | 64 | 31 |
| Male, % | 148 (61.4%) | 3 (2.2%) | 3 (8.8%) | 3 (2.7%) | 8 (12.5%) | 8 (25.8%) |
| Age | 66 (53 to 73) | 37 (29 to 46) | 59 (50 to 65) | 36 (29 to 46) | 48 (38 to 55) | 58 (53 to 62) |
| AFP (ng/ml) | 10.3 (4.1 to 598.3) | 3.0 (2.2 to 4.6) | 4.9 (3.3 to 8.0) | 3.3 (2.2 to 4.9) | 3.5 (2.1 to 6.0) | 4.2 (2.9 to 5.7) |
| PIVKA-II (mAU/ml) | 3227.0 (208.0 to 30577.5) | 42.5 (33.0 to 60.8) | 34.0 (30.0 to 46.3) | 37.0 (30.0 to 50.0) | 34.0 (29.3 to 46.8) | 34.0 (29.0 to 40.0) |
| ASAP | 2.38 (-0.10 to 4.90) | -2.65 (-3.24 to -2.16) | -1.64 (-2.25 to -0.89) | -2.79 (-3.31 to -2.19) | -2.17 (-2.91 to -1.35) | -1.78 (-1.96 to -1.03) |
| **Stages, %** |  |  |  |  |  |  |
| Early (BCLC 0-A) | 68 (28.2%) | NA | NA | NA | NA | NA |
| Intermediate (BCLC B) | 118 (49.0%) | NA | NA | NA | NA | NA |
| Advanced (BCLC C-D) | 48 (20.0%) | NA | NA | NA | NA | NA |
| Unknown | 7 (2.9%) | NA | NA | NA | NA | NA |
| **Etiology, %** |  |  |  |  |  |  |
| HBV | 11 (4.6%) | NA | NA | NA | NA | NA |
| HCV | 2 (0.8%) | NA | NA | NA | NA | NA |
| NAFLD | 80 (33.2%) | NA | NA | NA | NA | NA |
| ALD | 16 (6.6%) | NA | NA | NA | NA | NA |
| Others | 132 (54.8%) | NA | NA | NA | NA | NA |

Abbreviations: alpha-fetoprotein (AFP); protein induced by vitamin K absence-II (PIVKA-II); age, sex, AFP, and PIVKA-II (ASAP); Barcelona clinic liver cancer staging (BCLC); hepatitis B virus (HBV); hepatitis C virus (HCV); alcoholic liver disease (ALD); non-alcoholic fatty liver disease (NAFLD); focal nodular hyperplasia (FNH).

**Table S7. Cohort information of pre-HCC patients as well as early-stage HCC and cirrhotic individuals.**

| Variables | Control (cirrhotic) | Pre-HCC (cirrhotic) | Early-stage HCC (cirrhotic) |
| --- | --- | --- | --- |
| Number, n | 213 | 88 | 170 |
| Male, % | 121 (56.8%) | 71 (80.7%) | 123 (72.4%) |
| Age | 63 (56 to 68) | 68 (58 to 75) | 67 (60 to 74) |
| AFP (ng/ml) | 3.1 (2.3 to 4.9) | 7.1 (4.0 to 17.3) | 6.5 (3.5 to 17.3) |
| PIVKA-II (mAU/ml) | 28.0 (24.0 to 57.5) | 72.0 (42.0 to 157.0) | 153.0 (61.5 to 455.8) |
| AFP-L3(%) | 0.5 (0.5 to 5.5) | 5.5 (0.5 to 9.9) | 6.0 (0.5 to 10.0) |
| GALAD | -2.71 (-3.53 to -1.87) | -0.63 (-2.07 to 0.61) | -0.24 (-1.37 to 1.79) |
| ASAP | -1.12 (-1.63 to -0.54) | 0.29 (-0.31 to 0.93) | 0.40 (-0.29 to 1.65) |
| Time to HCC diagnosis (months)  **Stages, %** | NA | 13 (3.4) | NA |
| Early (BCLC 0-A) | NA | 60 (68.2%) | 170 (100%) |
| Intermediate (BCLC B) | NA | 16 (18.2%) | 0 |
| Advanced (BCLC C-D) | NA | 12 (13.6%) | 0 |
| Unknown | NA | 0 | 0 |
| **Etiology, %** |  |  |  |
| HBV | 0 | NA | 2 (1.2%) |
| HCV | 20 (9.4%) | NA | 18 (10.6%) |
| NAFLD | 119 (55.9%) | NA | 74 (43.5%) |
| ALD | 44 (20.7%) | NA | 46 (27.1%) |
| Others | 30 (14.1%) | NA | 30 (17.6%) |

Abbreviations: alpha-fetoprotein (AFP); lectin-reactive alpha-fetoprotein (AFP-L3); protein induced by vitamin K absence-II (PIVKA-II); Des-gamma-carboxy prothrombin (DCP); age, sex, AFP, and PIVKA-II (ASAP); Gender, Age, AFP-L3, AFP, DCP (GALAD); Barcelona clinic liver cancer staging (BCLC); hepatitis B virus (HBV); hepatitis C virus (HCV); alcoholic liver disease (ALD); non-alcoholic fatty liver disease (NAFLD).
