# Supplementary material for: Validation and optimization of AFP-based biomarker panels for early HCC detection in Latin America and Europe
Source: Hepatol Commun. 2023 Sep 15;7(10):e0264. doi: 10.1097/HC9.0000000000000264 (PMC10503685; doi:10.1097/HC9.0000000000000264)

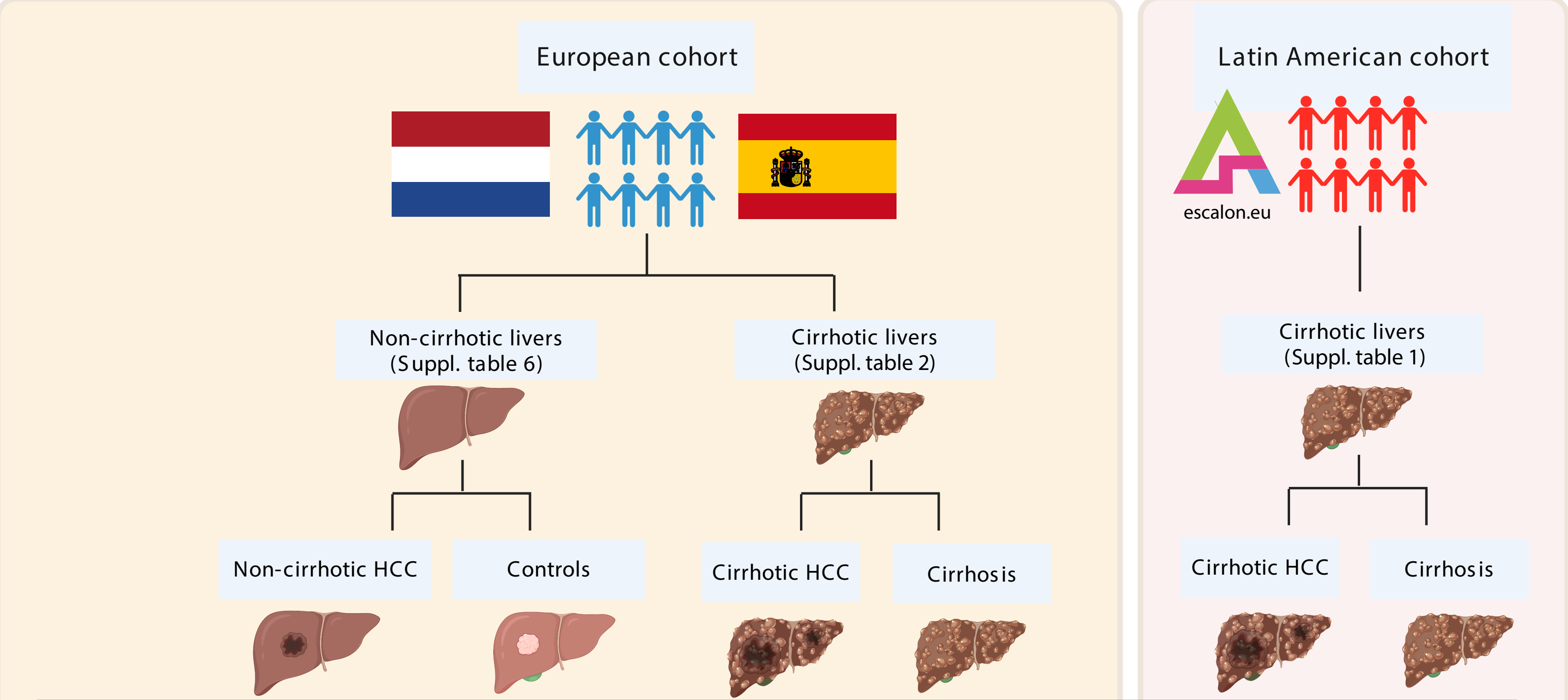

	HCC	Controls	HCC	Cirrhosis	HCC	Cirrhosis
GALAD	NA	NA	161	127	116	158
ASAP	241	378	556	191	188	393
GALAD/ASAP	NA	NA	116	66	113	155

Very early HCC detection cohort (Suppl. table 7)

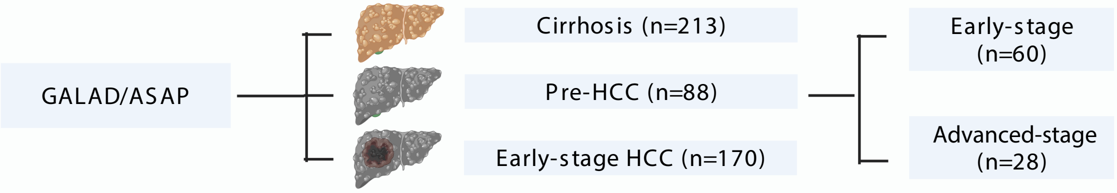

Supplement: Supplementary file 2 [file hc9-7-e0264-s002.pdf]
