# Supplementary material for: Validation and optimization of AFP-based biomarker panels for early HCC detection in Latin America and Europe
Source: Hepatol Commun. 2023 Sep 15;7(10):e0264. doi: 10.1097/HC9.0000000000000264 (PMC10503685; doi:10.1097/HC9.0000000000000264)
Supplement: Supplementary file 3 [file hc9-7-e0264-s003.pdf]

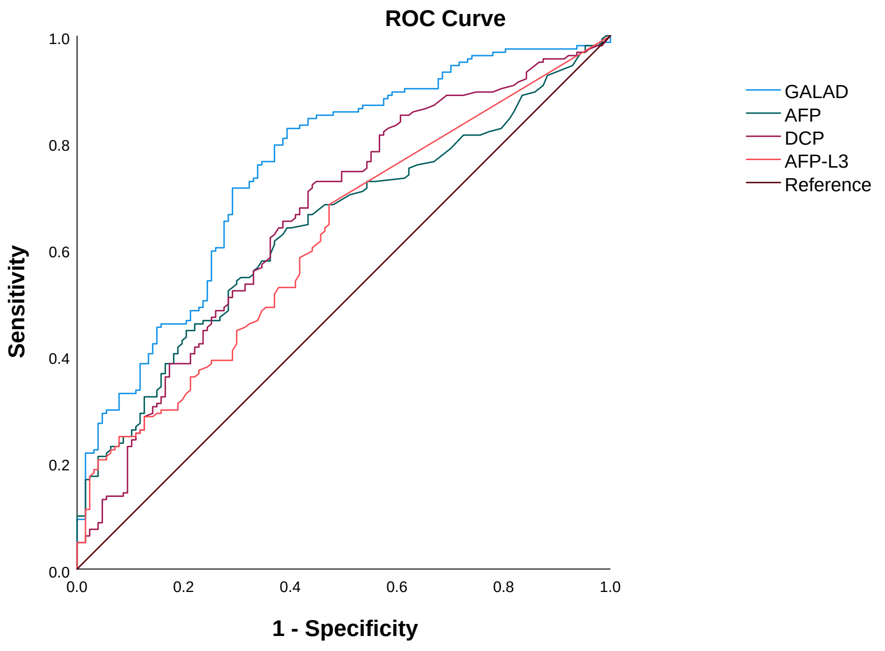

Performance of the GALAD score and its individual biomarkers in European patients. The GALAD score achieved higher AUC than the individual biomarkers AFP, DCP (PIVKA-II), and AFP-L3 in the European cohort (N=288).
