# Supplementary material for: Validation and optimization of AFP-based biomarker panels for early HCC detection in Latin America and Europe
Source: Hepatol Commun. 2023 Sep 15;7(10):e0264. doi: 10.1097/HC9.0000000000000264 (PMC10503685; doi:10.1097/HC9.0000000000000264)
Supplement: Supplementary file 4 [file hc9-7-e0264-s004.pdf]

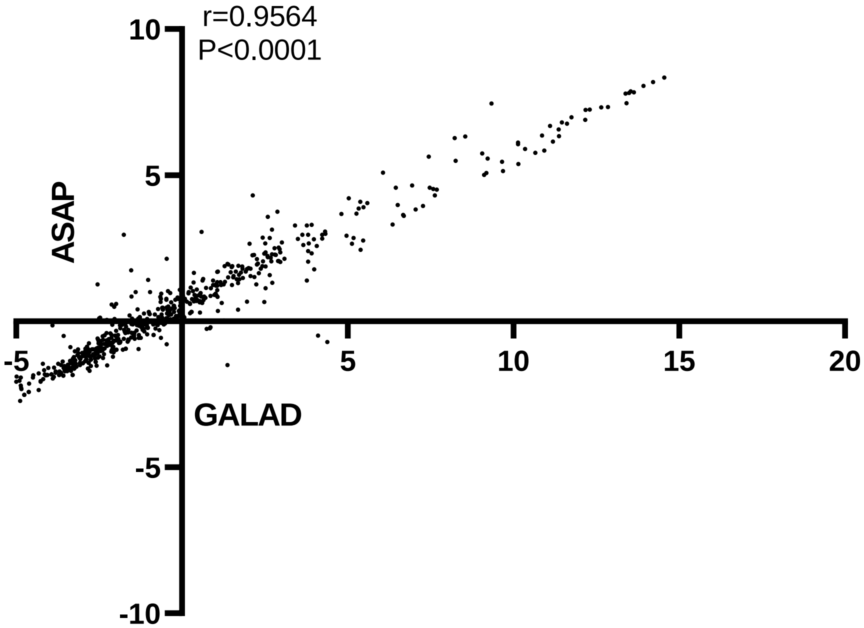

Association between GALAD and ASAP. ASAP showed a near-perfect correlation with GALAD using the Spearman's rank for correlation testing.
